# Dysregulated Neurotransmission and the Role of Viruses in Alzheimer’s Disease

**DOI:** 10.1021/acschemneuro.4c00763

**Published:** 2025-03-06

**Authors:** Katherine Bovis, Martha Davies-Branch, Philip J. R. Day

**Affiliations:** † Division of Evolution, Infection & Genomic Sciences, University of Manchester, Manchester M13 9PL, U.K.; ‡ Manchester Institute of Biotechnology, University of Manchester, Manchester M1 7DN, U.K.

**Keywords:** Alzheimer’s disease, Neurotransmission, HIV-1, Glutamate, HSV-1, l-Arginine

## Abstract

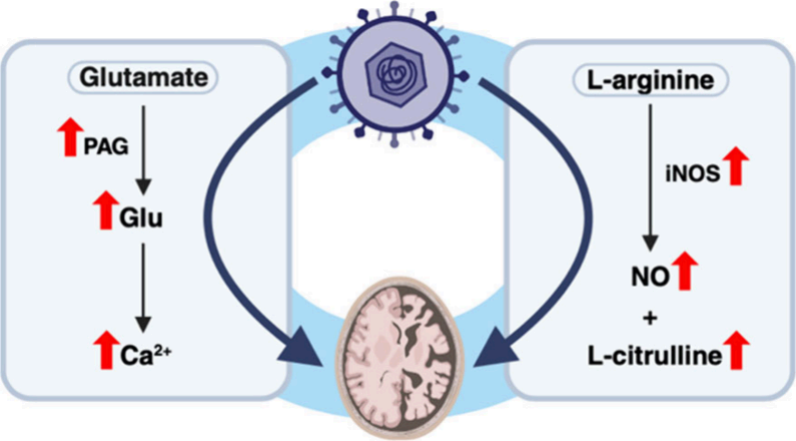

The causes of neurodegeneration remain elusive. There is growing evidence linking viral infection to dysregulated neurotransmission as a causative factor in Alzheimer’s disease. Studies suggest that viral infection may result in dysregulated glutamatergic and l-arginine/NO neurotransmission that can initiate neurodegeneration and neuroinflammation within AD. This involves viral infection (HIV-1/HSV-1) altering glutamate biosynthesis and receptor activation resulting in excessive influxes of glutamate and subsequent dysregulation of Ca^2+^ influx that all contribute to reduced dendrite growth and tau phosphorylation. For l-arginine/NO neurotransmission, the mechanism derives from the “protective” antiviral mechanisms of NO that correlate with pathologies such as β-amyloid peptide accumulation and functional degeneration of hippocampal neurons, respectively. More research is required to underpin the direct mechanisms that viruses might impact to induce specific pathologies.

## Introduction

Neurodegenerative diseases (NDDs) involve progressive loss of neuronal connections, in the requirement for novel disease modifying therapies (DMTs) targeting NDDs such as Alzheimer’s disease (AD) and multiple sclerosis (MS) is apparent given that collectively these diseases contributed to ∼1.82 million worldwide deaths in 2019.^1,2^ Anticipated to only increase due to the aging world population, global NDD prevalence will continue to increase, given the current poor comprehension of disease pathology, limited biomarkers and effective related DMTs.

AD is one of the most prevalent NDDs and is characterized by presence of amyloid oligomers within the brain where amyloid beta (Aβ) deposits are recognized to dysregulate neuronal cell homeostasis resulting in cellular apoptosis.^3^ As a result, most research is focused on investigating the “amyloid hypothesis” of AD development and has recently resulted in the development of novel immunotherapies targeting amyloid fibers employing Lecanemab and Donanemab.^4^ This is a promising example for novel NDD early stage therapeutics; however, the causative pathogenesis remains evasive.

The role of viruses in NDD development is not a recent concept with M. J. Ball^5^ proposing the viral hypothesis where herpes simplex virus-1 (HSV-1) infection in AD led to dysregulated neurotransmission and thus neurodegeneration. To date, a growing body of research has supported this hypothesis and the potential role of a wide range of viruses in NDD.^6^ For example, a study by Levine and colleagues^7^ surveyed cross-sectional and longitudinal associations between NDDs and viral exposures through resources obtained from the FinnGen project and UK biobank. This study identified 22 viruses associated with increased NDD risk, where the highest hazard ratio (30.72) was detected between viral induced encephalitis and AD.

To date there are multiple studies supporting a viral role in AD development such as the proposal that there is an infectious origin of AD where Aβ functions as an antimicrobial peptide (AMP) and viral infection therefore “seeds” aggregation of Aβ.^8^ Moreover, it has also been suggested that virally encoded MicroRNAs (miRNAs) have a role in manipulating host cellular gene expression, thus dysregulating neurodegenerative risk pathways, and triggering AD development.^9^ However, despite mounting evidence, there are challenges associated with validating a viral role in NDD development. A major caveat is the host immunological response to infection presenting a high pathogen-related response background, plus the latent-lytic process and reactivation^9^ of many viruses that add further background molecular signatures that constrains the application of standard analytical detection methods.

In this review, we will focus on the potential role of viruses in the dysregulation of neurotransmission that may lead to AD development with a specific focus on the impact of Human Immunodeficiency Virus (HIV-1) and HSV-1 viral infection on neuroregulatory pathways including glutamate and l-arginine signaling, and the associated role of calcium flux.

## Glutamate Neuroregulatory Pathway

**Figure 1:**
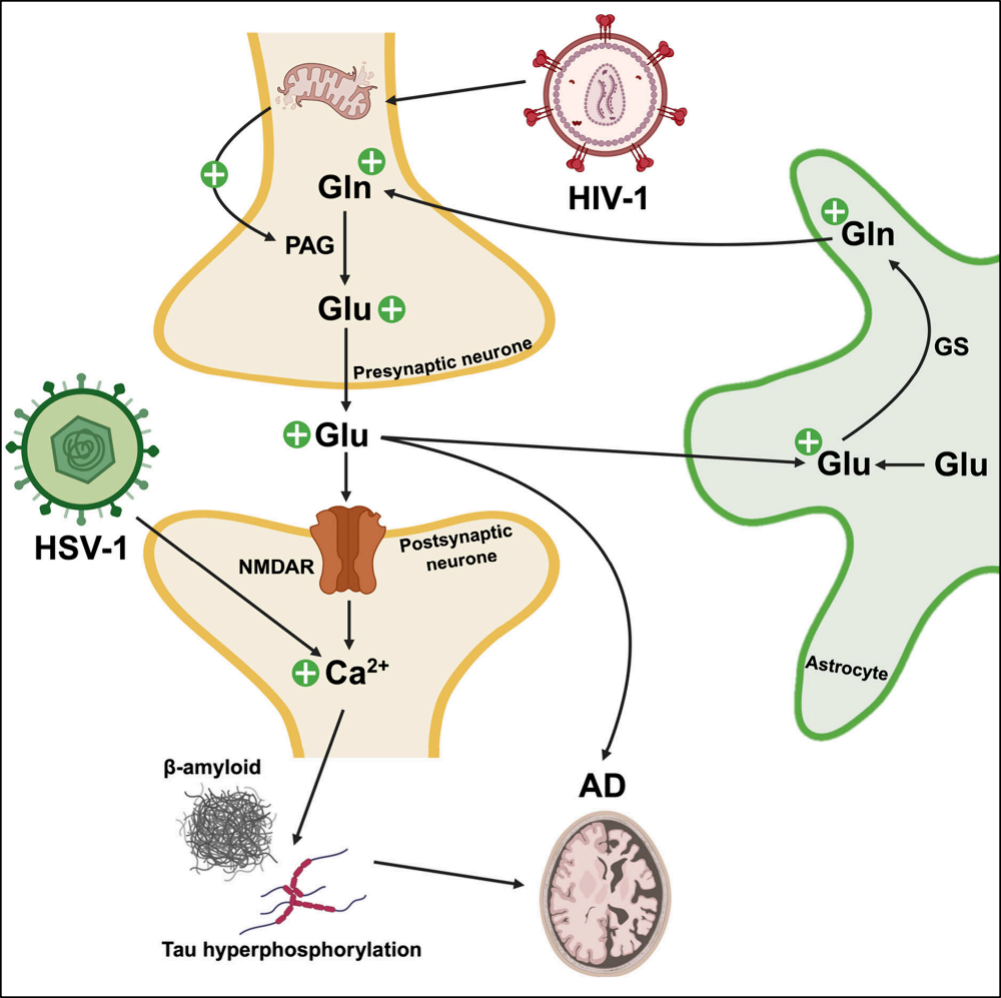
Viral engagement of glutamate neuroregulatory pathway in Alzheimer’s disease viral infection has been linked to dysregulation of glutamate neuroregulatory pathways and suggested to contribute to Alzheimer’s disease (AD) pathology. Altered biosynthesis: human immunodeficiency virus (HIV-1) was shown to destabilize mitochondrial membrane through infection of neurons and led to cytosolic release of phosphate-activated glutaminase (PAG). Increased PAG availability directly impacts glutamate (Glu) biosynthesis and results in increased conversion of glutamine (Gln) to Glu. Increased conversion of Glu to Gln via glutamine synthetase (GS) is also observed. Increased levels of Glu may directly impact neurodegeneration in AD such as memory deficits, impaired long-term potentiation (LTP) and reduced dendrite growth. Receptor Activation: Increased biosynthesis of Glu, as a result of HIV-1 infection, can lead to hyperexcitation of *N*-methyl-d-aspartic acid receptors (NMDARs) on postsynaptic neurons causing heightened influxes of Ca^2+^. HSV-1 infection is also suggested to increase intraneuronal Ca^2+^ levels. Excessive influx of Ca^2+^ has been associated with AD pathogenesis through tau hyperphosphorylation, synaptic dysfunction and increased intracellular concentrations of β-amyloid.

Glutamatergic neurotransmission is significantly dysregulated in NDDs such as AD and this has been explored in several studies (Figure 1).^10^ While the underlying mechanisms behind the dysregulation are largely unknown it has been suggested that viral infection and the resulting immune response play a vital role, indirectly promoting neurodegenerative processes.^12^

Within the CNS, glutamate (Glu) is the most common neurotransmitter where it mediates five major cortical pathways.^11^ Processes that rely on appropriate regulation of Glu include learning, memory, pain, and synaptogenesis where dysfunction promotes neuronal death and degeneration.^13^ Glutamatergic neurotransmission also involves numerous receptors, including a group of ionotropic glutamatergic receptors known as *N*-methyl-d-aspartic acid receptors (NMDARs). Binding of Glu at postsynaptic neurons generates an influx of calcium ions (Ca^2+^), increasing intracellular calcium concentrations. Intracellular neuronal Ca^2+^ plays an activating role in many signaling pathways and processes, e.g., neurite spine formation.^14^ Therefore, Ca^2+^ dysregulation can cause a host of abnormalities, including neuronal atrophy and neurodegeneration.

High levels of Glu have been documented in patients with AD.^15^ Maderia et al.^16^ investigated changes in cerebrospinal fluid (CSF) Glu levels in age matched patients with probable AD compared to controls. It was found that mean CSF Glu levels were significantly higher in patients with probable AD suggesting that glutamatergic neurotransmission is hyperregulated in AD pathology. The effects of dysregulated Glu levels in AD development are still under investigation; however, high levels of Glu within the brain have been linked to impaired long-term potentiation (LTP) and memory deficits.^17^ For example, Monnerie et al.^18^ have demonstrated that large increases in Glu release can result in neurodegeneration. In this study, the effect of Glu on dendrite growth was examined where reduced dendrite growth was observed in cortical neurones exposed to excess Glu. Therefore, the excess levels of Glu within the brain may contribute to the development of NDD and thus AD.

One mechanism underpinning excess Glu levels in AD is the involvement of viral infection and its exploitation of glutamatergic neurotransmission in the NDD. For example, several neurotropic viruses, notably HIV-1 and associated proteins (including Tat) have been found to disrupt glutamatergic neurotransmission.^19^ HIV-1^+^ patients displaying neurological symptoms present with elevated levels of Glu-responsive factors were a distinct group when compared to both patients without these symptoms and healthy controls.^20^ The mechanisms viruses, such as HIV-1, may exploit to influence glutamatergic neurotransmission is still not completely understood, although multiple mechanisms of action have been proposed, including producing excessive release of Glu into the synaptic cleft, causing excitotoxicity of the glutamatergic neurotransmission system. This theory is supported by increased levels of glutamate in the CSF of HIV-1 patients, which is positively correlated with dementia and brain atrophy.^21^ Furthermore, there are several ways that viruses may enact these effects, including altered biosynthesis and receptor activation (Figure 1). Viruses (HIV-1) have been shown to affect Glu biosynthesis through various mechanisms. Within the central nervous system (CNS), Glu biosynthesis occurs in astrocytes and neurons, requiring their coordination for successful synthesis.^22^ De novo synthesis of Glu occurs exclusively in astrocytes due to the unique localization of pyruvate carboxylase (PC) and glutamine synthetase (GS) in these cells. In astrocytes, Glu is produced via PC and then converted to Gln using GS. Once released into the synaptic cleft for receptor activation, excess Glu can be reuptaken by astrocytes and converted back to Gln via GS. Any nonreactive Gln is then released into the extracellular space where presynaptic Glu neurons use it to convert into Glu via phosphate-activated glutaminase (PAG).^23^ PAG is also expressed in astrocytes. Persistent activation of resident immune cells, such as microglia and astrocytes, due to viral infection has been shown to increase Glu biosynthesis,^24^ where for example the HIV-1 viral protein Tat has been shown to activate macrophages and microglia.^25,26^ These studies led to a proposal that viruses, such as HIV-1, dysregulate biosynthesis by altering levels of GS and PAG. In this context HIV-1 has been shown to cause mitochondrial membrane destabilization upon neuronal infection, leading to the cytosolic release of PAG.^13^ Increased PAG availability would then contribute to further Glu accumulation, potentially causing damage.

Viral influence on glutamatergic neurotransmission is also achieved through effects imposed on receptor activation. Glu binding to NMDARs on postsynaptic neurons generating influx of Ca^2+^ has been discussed, and excessive Ca^2+^ influx, as a result of Glu induced hyperexcitation of NMDARs, causes various aberrant processes in the CNS such as overactivation of protein kinases involved in tau hyperphosphorylation, inappropriate ROS production, and neuronal collapse.^27^ For example, Acuña-Hinrichsen and colleagues^14^ observed the structural collapse of HSV-1-infected neurons. Since neuronal calcium plays a role in neurite spine formation, abnormal Ca^2+^ signals can trigger actin cytoskeleton rearrangement in postsynaptic spines causing altered synaptic morphology and thus structural neuritic damage.^14^ In the context of AD pathophysiology, dysregulated calcium homeostasis has been documented. Excessive Ca^2+^ fluxes as a result of overactivation of NMDAR by Glu has been shown to lead to synaptic dysfunction and tau phosphorylation, a hallmark of AD.^28^ The FDA approved drug Memantine acts as a noncompetitive open channel NMDAR blocker and is prescribed to AD patients for memory preservation. Use of Memantine for treatment of AD directly shows that abnormal Ca^2+^ flux resulting from dysregulated glutamatergic transmission is involved in AD development.^29^ Links between viral infection (HSV-1), dysregulated Ca^2+^ signaling, and AD have also been documented. This includes a study by Piacentini et al.^30^ aiming to explore the relationship between infection with HSV-1 and AD development through investigating changes in electrophysiology properties in rodent cortical neurones. Overall, following HSV-1 infection, there was dysregulation in intracellular Ca^2+^ signaling, which was shown to increase intracellular accumulation of Aβ. Therefore, HSV-1 infection may induce abnormal Ca^2+^ levels as a result of dysregulated glutamatergic neurotransmission and contribute to AD pathogenesis.

## l-Arginine Neuroregulatory Pathway

**Figure 2:**
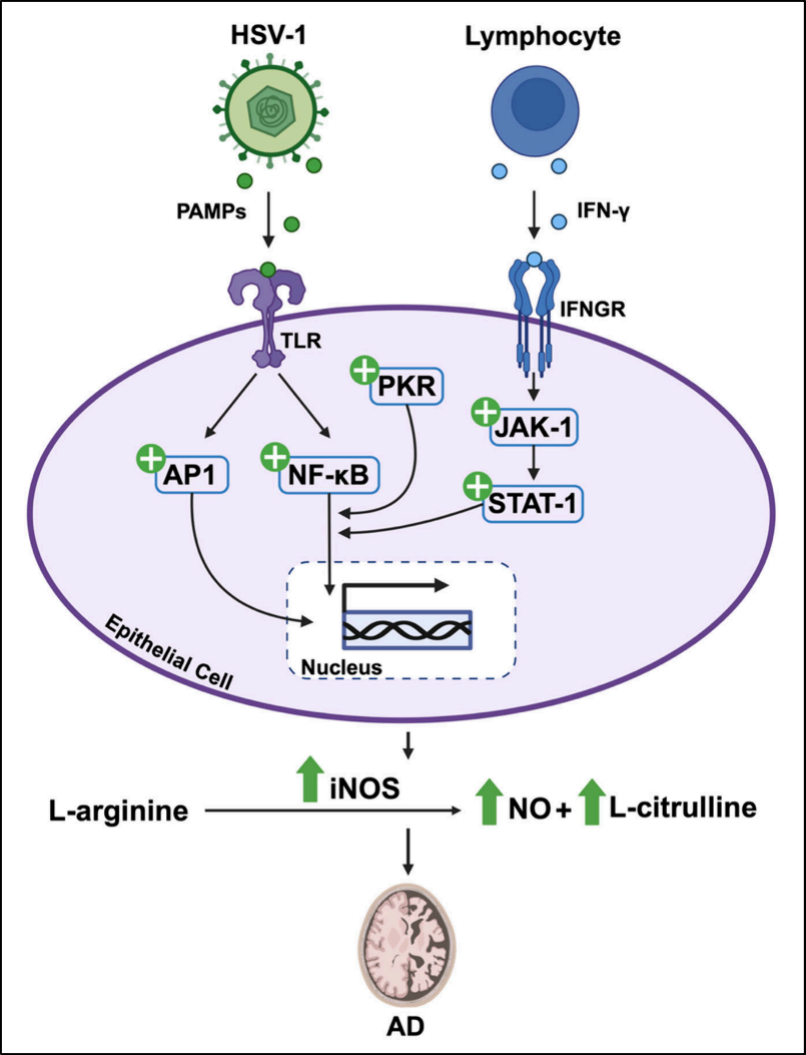
Viral engagement of l-arginine neuroregulatory pathway in Alzheimer’s disease viral infection has been linked to dysregulation of l-arginine neuroregulatory pathways and suggested to contribute to Alzheimer’s disease (AD) pathology. Endogenous nitric oxide (NO) production is increased in a “protective” response to viral infection, such as Herpes Simplex Virus-1 (HSV-1) infection, by three antiviral mechanisms initiated by inducible nitric oxide synthase (iNOS) upregulation. Mechanism 1: Toll-like receptors (TLRs) expressed on epithelial cells detect pathogen-associated molecular patterns (PAMPs) that in the context of viral infection are typically components of viral envelopes or viral nucleic acid. Detection of PAMPs then triggers activation of signaling pathways that lead to activation of two transcription factors, nuclear factor-κB (NF-κB) and activator protein 1 (AP1). Activated NF-κB and AP1 then enter the nucleus and cause upregulation of iNOS transcription. Mechanism 2: 24–26 h postviral infection, interferon gamma (IFN-γ) produced by activated immune cells induces phosphorylation and activation of Janus kinase-1 (JAK-1). JAK-1 initiates the phosphorylation and activation of signal transducer and activator of transcription 1 (STAT-1) that subsequently leads to upregulation of iNOS via NF-κB. Mechanism 3: Activated by viral infection, double-stranded RNA dependent protein kinase (PKR) also triggers upregulation of iNOS through interactions with the NF-κB pathway. iNOS upregulation from mechanisms 1–3 results in increased conversion of l-arginine to NO and l-citrulline. Higher concentrations of NO can have negative effects and contribute to AD pathology through colocalization with β-amyloid peptide accumulation, nitrergic and glycation linked cellular stress and thus neurodegeneration.

Viral infection may exploit and dysregulate l-arginine-related neurotransmission, which may in turn promote neurodegeneration (Figure 2). The tightly regulated metabolism of l-arginine, a semiessential proteinogenic amino acid, is key for production of several bioactive molecules that are important for neurotransmission.^31,32^ For example, l-arginine is involved in two major metabolic pathways: the dominant nitric oxide synthase (NOS) pathway and the arginase pathway. The arginase pathway involves the conversion of l-arginine into l-ornithine and urea, catabolized by the enzyme arginase I/II (ArgI/II). The NOS pathway involves the conversion of l-arginine into l-citrulline and nitric oxide (NO), catabolised by the enzyme NOS.^31^ NO goes on to play essential roles as a signal transduction molecule and important effector within a vast range of physiological processes such as immune responses, vasodilation, and neurotransmission.^32,33^

In the context of neurotransmission, NO acts as a gaseous neurotransmitter via stimulation of the cyclic guanosine monophosphate (cGMP) pathways. These pathways are involved in excitatory neurotransmission and are known to inhibit synaptic transmission.^34^ Specifically within the brain, NO is endogenously generated as a free radical and is derived from three different NOS isoforms.^35,36^ These include (1) the constitutively expressed endothelial NOS (eNOS) and (2) neuronal NOS (nNOS), both of which are important in maintenance of cerebral blood flow, memory, and synaptic plasticity. The third isoform, (3) inducible NOS (iNOS), is expressed only when cells are stimulated, often by immune and inflammatory responses to an invading pathogen.^37^ Within the normal brain, eNOS and nNOS are constitutively expressed whereas in the context of iNOS, its increased expression within the CNS is proposed to be pathological.^38^ Thus, iNOS could play a key role in the relationship between viral infection and neuronal function.

The outcome of the neurotransmitter actions of NO varies depending on its concentration. For example, endogenous NO production is often considered a paradoxical situation where despite having many beneficial physiological roles (i.e., control of vascular tone), high concentrations of NO, resulting from overexpression of iNOS, can result in damaging effects within the brain such as neurodegeneration.^39,34^ For example, it has been shown that inhibition of iNOS to reduce NO production protects against Aβ-induced neurotoxicity.^40^ This places emphasis on the importance of tight regulation of NOS signaling where there is a delicate distinction between beneficial and damaging concentrations of NO^37^ and offers a possible viral mediated NDD mechanism to exploit and cause damage to neurons.

In response to infection from viruses such as HSV-1, endogenous NO production is upregulated via three antiviral mechanisms all initiated by iNOS upregulation as proposed by Sodano and colleagues^41^ (Figure 2). Overall, viral presence causes upregulation of iNOS resulting in increased conversion of l-arginine to l-citrulline, producing high concentrations of NO, which has antiviral action. However, as previously stated, high levels of NO can be detrimental and result in neurotoxicity that can lead to neurodegeneration within NDDs such as AD. Cymerys and colleagues^42^ explored the antiviral roles of NO during infection wherein they investigated NO effects and nonapoptotic Fas signaling in various HSV-1 models. Here, they showed nontoxic concentrations of NO decrease HSV-1 replication in mouse neuronal cultures demonstrating a possible antiviral role of NO. However, they also showed that in both in vivo and in vitro HSV-1 infected neurones, NO production colocalized with β-amyloid peptide accumulation where this was reversed by treatment with aminoguanidine, an iNOS inhibitor. This study provides evidence indicating that protection of antiviral mechanisms involving upregulation of iNOS and subsequent increased NO production may in turn promote off-target neurodegenerative processes within AD.

Microglia are involved in first line defense in response to HSV-1 infection and are a major source of iNOS where these immune cells increase NO production to avoid cell death during viral infection.^43,42^ Microgliosis has also been found associated with upregulation of iNOS and decreased Arg1 expression in response to prion infection within mouse models.^44^ This mirrors the action of microglia observed in AD pathogenesis where their iNOS production is upregulated in response to Aβ-associated inflammation suggesting a harmful cycle of neuroinflammation in response to increased iNOS levels.^38^

The role of dysregulated NO in triggering neurodegenerative processes within AD has also been investigated. Bourgognon and colleagues^45^ investigated the effects of excessive NO production in AD and prion disease where they hypothesized NO exerts post-translational protein modifications such as induction of protein nitrotyrosination. The effects of protein nitrotyrosination have previously been demonstrated in the context of β-amyloid (Aβ) oligomers where NO was detected to target Aβ, nitrating the protein at tyrosine 10 (3NTyr^10^-Aβ). This nitration subsequently accelerated Aβ aggregation and was identified within the Aβ plaques of AD brains and APP/PS1 mice.^46^ Bourgognon and colleagues^45^ showed that functional degeneration of hippocampal neurones in prion-infected mice was prevented by daily in vivo injections of L-NAME, a NOS inhibitor. It was concluded that the observed reduction in neurodegeneration resulted from reduced 3-nitrotyrosination of an enzyme involved in disease-associated glycation known as triose-phosphate isomerase. Overall, this study suggests that by inhibiting neuroinflammatory NO signaling, neurodegeneration is subsequently slowed and nitrergic and glycation linked cellular stress is decreased.

Moreover, a metabolomics study in 2017 investigating the changes in urine metabolites between APP/PS1 mouse models and wild type controls^47^ revealed 24 differential expressed metabolites. One of the metabolites highlighted was 4-guanidinobutanoic acid, a downstream product of arginase metabolism.^48^ It was found that 4-guanidinobutanoic acid was upregulated in APP/PS1 mouse urine samples implying NO production may be dysregulated in these AD models and contribute to neurodegenerative processes within AD.^47^

## Summary

There is growing evidence to suggest viral infection may result in dysregulated glutamatergic and l-arginine/NO neurotransmission, which can successively trigger neurodegeneration and neuroinflammation within AD. This involves HIV-1/HSV-1 infection altering glutamate biosynthesis and receptor activation, resulting in excessive influxes of glutamate and subsequent dysregulation of Ca^2+^ influx, which all contribute to reduced dendrite growth and tau phosphorylation. In the context of l-arginine/NO neurotransmission, this manifests from the “protective” antiviral mechanisms of NO, which multiple studies have demonstrated to correlate with pathologies such as β-amyloid peptide accumulation and functional degeneration of hippocampal neurons, respectively. Despite the growing evidence linking viral infection to dysregulated neurotransmission as a causative factor in AD, more research is required to underpin the direct mechanisms viruses exert to induce specific pathologies.
